# Biodegradable polyester-based nano drug delivery system in cancer chemotherapy: a review of recent progress (2021–2023)

**DOI:** 10.3389/fbioe.2023.1295323

**Published:** 2023-11-01

**Authors:** Zongheng Wang, Miaomiao Xiao, Fangliang Guo, Yue Yan, Hong Tian, Qianshi Zhang, Shuangyi Ren, Liqun Yang

**Affiliations:** ^1^ Department of Gastrointestinal Surgery, The Second Affiliated Hospital of Dalian Medical University, Dalian, China; ^2^ Liaoning Research Institute of Family Planning (The Reproductive Hospital of China Medical University), Shenyang, China; ^3^ College of Kinesiology, Shenyang Sport University, Shenyang, China; ^4^ Department of Emergency, The Second Affiliated Hospital of Dalian Medical University, Dalian, China; ^5^ Department of Oncology, The 4th People’s Hospital of Shenyang, China Medical University, Shenyang, China; ^6^ Research Center for Biomedical Materials, Shengjing Hospital of China Medical University, Shenyang, China

**Keywords:** biodegradable polyester, drug delivery system, nanoparticles, cancer, chemotherapy

## Abstract

Cancer presents a formidable threat to human health, with the majority of cases currently lacking a complete cure. Frequently, chemotherapy drugs are required to impede its progression. However, these drugs frequently suffer from drawbacks such as poor selectivity, limited water solubility, low bioavailability, and a propensity for causing organ toxicity. Consequently, a concerted effort has been made to seek improved drug delivery systems. Nano-drug delivery systems based on biodegradable polyesters have emerged as a subject of widespread interest in this pursuit. Extensive research has demonstrated their potential for offering high bioavailability, effective encapsulation, controlled release, and minimal toxicity. Notably, poly (ε-caprolactone) (PCL), poly (lactic-co-glycolic acid) (PLGA), and polylactic acid (PLA) have gained prominence as the most widely utilized options as carriers of the nano drug delivery system. This paper comprehensively reviews recent research on these materials as nano-carriers for delivering chemotherapeutic drugs, summarizing their latest advancements, acknowledging their limitations, and forecasting future research directions.

## Graphical Abstract

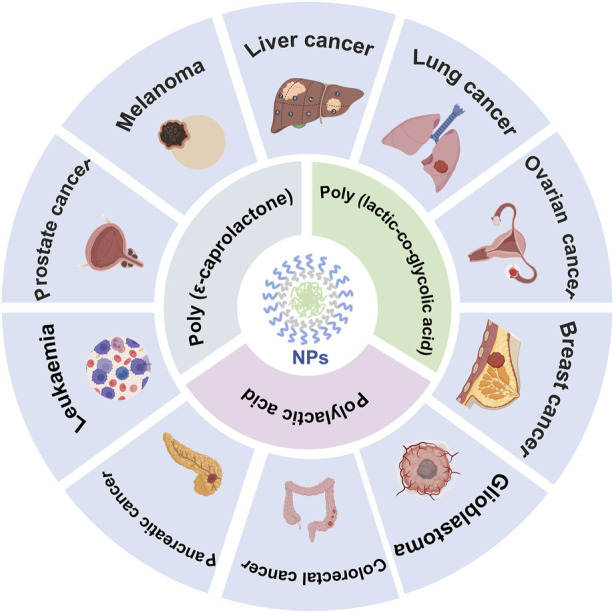

## 1 Introduction

**FIGURE 1:**
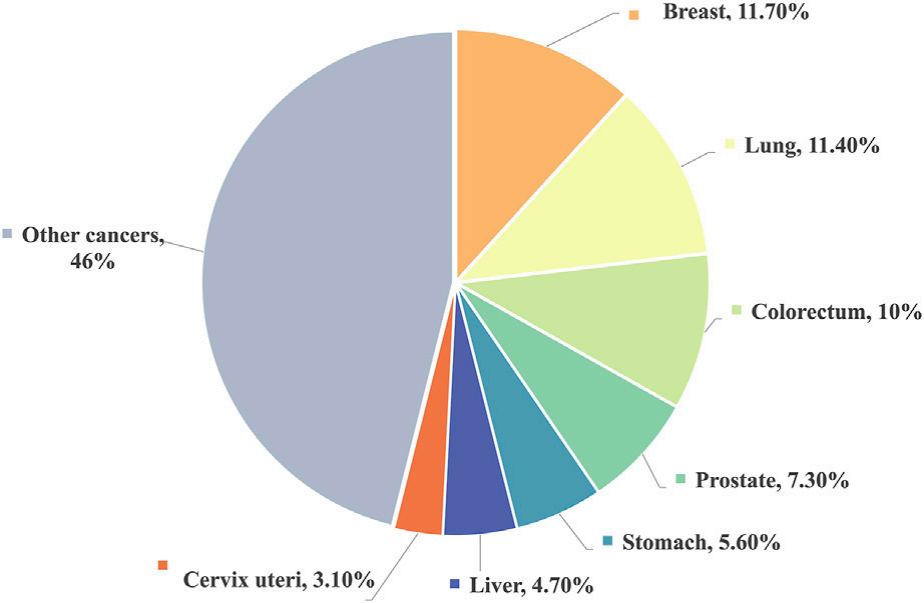
Estimated proportion of new cancer cases in 2020, World, both sexes, all ages.

Cancer is the second most prominent contributor to global mortality, trailing only behind cardiovascular diseases (Mattiuzzi and Lippi, 2019). Figure 1 illustrates data from the World Health Organization, revealing that breast cancer claimed the top spot for new cancer cases in 2020, closely followed by lung cancer (W.H. O, 2023). Based on statistics provided by the American Cancer Society, it was projected that the United States would witness 1,958,310 new cancer diagnoses and 609,820 cancer-related fatalities in 2023 (Siegel et al., 2023). Undoubtedly, cancer exerts a substantial financial burden on healthcare systems worldwide, posing significant challenges to their fiscal resources and long-term viability (Qiu et al., 2021).

Current treatment modalities primarily encompass surgical procedures, chemotherapy, and immunotherapy. However, certain advanced-stage patients may not qualify for surgical interventions, and even post-surgery, some may encounter relapses (Almeida et al., 2019). Immunotherapy, while available, remains accessible to only a limited fraction of patients and carries severe side effects, including autoimmune reactions and non-specific inflammation (Riley et al., 2019). Chemotherapy, administered before and after tumor removal, serves the dual purpose of facilitating surgical procedures and preventing the resurgence of residual cancer cells. It enjoys widespread utilization and is indispensable in cancer treatment (Hellmann et al., 2016). Prominent chemotherapy agents such as 5-fluorouracil (5-FU), paclitaxel (PTX), doxorubicin (DOX), and cis-diamminedichloro-platinum (CDDP) find extensive clinical application and yield favorable treatment outcomes (Roth and Ajani, 2003). Nevertheless, these chemotherapy drugs have limitations, including restricted bioavailability, suboptimal tissue penetration, the absence of specific targeting ligands, and the necessity for frequent administration (Kozovska et al., 2014; Chou et al., 2020). Furthermore, although some patients exhibit an initial positive response to treatment, they may subsequently develop resistance to chemotherapy, culminating in tumor recurrence (Biller and Schrag, 2021; Xiao et al., 2021; Yoon et al., 2021).

Hence, the quest for an improved drug delivery system has piqued the interest of scholars, with nanoparticles based on biodegradable polyesters emerging as a focal point of attention (Cheng and Pun, 2015). Biodegradable polyesters are polymeric materials that boast environmentally friendly attributes (Gross and Kalra, 2002). Additionally, they exhibit commendable biocompatibility and can decompose into small molecule byproducts within the human physiological milieu. Several have secured approval from the U.S. Food and Drug Administration for diverse clinical applications in drug delivery systems, including PCL (Malikmammadov et al., 2018), PLA (Williams, 2007; Pandey et al., 2015) and PLGA (Sonam Dongsar et al., 2023), among others. Their structures are outlined in Table 1 (Washington et al., 2017).

**TABLE 1:** The structures of the polymers.

Polymer	Structure	T_g_ (^o^C)	T_m_ (^o^C)
PCL	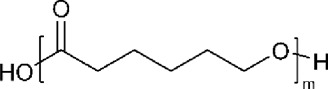	−60	60
PLGA	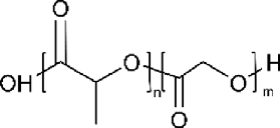	35–60	120–200
PLA	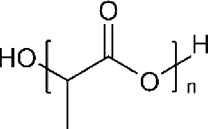	60–65	150–160

On the one hand, these nanoparticles can traverse the endothelial barriers of the spleen and liver; on the other hand, they leverage the enhanced permeability and retention (EPR) effect to passively accumulate at tumor sites (Zhang et al., 2014; Asadi et al., 2017). They possess robust drug-loading capabilities, facilitate optimal intracellular uptake, and harness the enhanced permeability and retention phenomena (Bae et al., 2011; Yao et al., 2020). In principle, nanomaterials offer a hydrophobic core for encapsulating drugs, enhancing their stability in the bloodstream (Gupta et al., 2021). Moreover, they can be customized by incorporating various functional groups to modulate their functions within the body (Esfandyari-Manesh et al., 2016).

To enhance the active targeting of the delivery system, researchers usually employed cancer cell-specific ligands as targets. These ligands enable easy entry into cancer cells through receptor-mediated transcytosis, circumventing sole reliance on the EPR effect. Such ligands encompass peptides, polysaccharides, antibodies, and more (Sun et al., 2018).

This review presents an overview of the researches conducted over the past 3 years concerning biodegradable polyesters for the delivery of cancer chemotherapeutic drugs. Our focus centers on the extensively studied PCL, PLGA, and PLA, summarizing the latest advancements, recognizing their limitations, and shedding light on future research trends.

## 2 Biodegradable polyester-based drug delivery systems

### 2.1 Poly (ε-caprolactone) (PCL)

PCL is a biodegradable and biocompatible semi-crystalline linear aliphatic polyester (Khan et al., 2017). It is non-toxic, biodegradable, and biocompatible attributes, which is used in a wide range of bio-applications (Bhadran et al., 2023; Murab et al., 2023). The details about the applications of PCL in cancer chemotherapy are shown in Table 2.

**TABLE 2:** PCL-based nano drug delivery systems investigated for treating cancers.

Author	Year	Nanoparticles system	Size (nm)	Encapsulation efficiency (%)	Drug loading (%)	IC50	The blood circulation time	Type of tumor	Chemotherapy drug	Reference
Nasrullah Jan et al	2021	Cytarabine-PCL	120.5 ± 1.18–341.5 ± 3.02	41.31 ± 0.49–62.28 ± 0.39%	6–20	KG-1 cells, 48 h, 8.80 ± 0.48 μg/mL	—	Leukaemia and breast cancer	Cytarabine	Jan et al. (2021)
Safiullah Khan et al	2021	5-FU LPHNPs	174 ± 4–267 ± 2.65	92.87 ± 0.59–94.13 ± 0.77	6.25–12.5	HeLa cells, 47.34 μg/mL	—	Breast cancer	5-FU	Khan et al. (2021)
Hongdan Shen et al	2021	mPEG-b-PCL-DOX	300	—	4	HCT116 cells, 2.65 ± 0.29 μg/mL	—	Colorectal cancer	DOX	Shen et al. (2021)
Akanksha Behl et al	2022	PEG-PCL	128.66 ± 23	85.4	—	MCF-7 cells, 22 nM	—	Breast cancer	Gemcitabine	Behl et al. (2022)
Wufa Fan et al	2022	OPDEA-PCL	45.4	—	7.3	NCI-H520 cells, 48 h, 2.52/0.62 μg/mL (CDDP/PTX)	>24 h	Liver and lung cancer	CDDP/PTX	Fan et al. (2022)
De-Chao Yang et al	2022	mPEG-b-PCL	—	—	4	HepG2 cells, 1.89 nM	—	Liver and lung cancer	Camptothecin	Yang et al. (2022)
Jiajia Xiang et al	2022	OPDMA-PCL/OPDEA-PCL	29/25	82.3/80.8	15.7/14.9	HeLa cells, 1.15/1.38 μg/mL	>8 h	Breast cancer	DOX	Xiang et al. (2022)
Ziting Zhang et al	2022	RSV-NPs@RBCm	160.91 ± 0.63	45.25	7.54	HCT116 cells 23.65 ± 3.21 μg/mL	>48 h	Colorectal cancer	Resveratrol	Zhang et al. (2022a)
Qianqian Zhang et al	2022	Spm-PEG-PCL-DOX	69.3 ± 3.4	110.91 ± 9.68	13.9 ± 0.6	HCT116 cells 35.42 ± 1.16 μg/mL	—	Lung cancer	DOX	Zhang et al. (2022b)
Kimiya Hasanbegloo et al	2023	chitosan (core)/PCL-chitosan (shell)	135 ± 45	72.1 ± 2.8	—	—	—	Breast cancer	PTX	Hasanbegloo et al. (2023)
Chae Eun Jin et al	2023	mPEG-b-PCL	33.1 ± 2.15	94.4 ± 4.14	2.61 ± 0.26	HeyA8 cells 75.8 nM	4 h	Ovarian cancer	PTX	Jin et al. (2023)
Yihong He et al	2023	ARV-DOX/cRGD-PEG-PCL	59.31	94	2.5	—	—	Colorectal cancer	DOX	He et al. (2023)

To harness the advantages of PCL in addressing the short half-life and limited bioavailability associated with intravenous drug administration, Jan et al. (2021) employed a nanoprecipitation method to create PCL nanoparticles loaded with arabinosylcytosine. They investigated the *in vitro* anti-cancer effects of these nanoparticles on KG-1 leukemia cells. The *in vitro* release experiments revealed an initial burst release followed by sustained release over 48 h. Additionally, cytotoxicity experiments demonstrated that the IC50 value of PCL nanoparticles was nearly two orders of magnitude lower than that of the pure drug injection. This underscored the effectiveness of arabinosylcytosine-loaded PCL nanoparticles as a drug carrier, effectively mitigating dose-related toxicity while offering a controlled release mechanism.

In contrast to free drugs, liposomes exhibit improved pharmacokinetics and enhanced biocompatibility. They can accommodate hydrophilic and lipophilic drugs and can be customized as needed. However, liposomes face limitations such as low drug loading, rapid release, leakage, and instability during storage. To address these issues, Khan et al. (2021) employed a nano-precipitation technique to create 5-fluorouracil-loaded lipid-polymer hybrid nanoparticles (LPHNPs). The IC50 values of free 5-FU and 5-FU LPHNPs were 60.78 μg/mL and 47.34 μg/mL for HeLa cells and 58.35 μg/mL and 43.33 μg/mL for MCF-7 cells, respectively. The reduced IC50 values of LPHNPs suggest they are more effective at killing cancer cells than free drugs. *In vitro* release studies showed an initial burst release of 40% in the first 9 h, followed by continuous release over 72 h.

Furthermore, Kimiya Hasanbegloo and colleagues conducted similar research. They loaded paclitaxel into liposomes and embedded them within PCL/chitosan nanofibers to enhance sustained paclitaxel delivery. Drug release experiments indicated a sustained release period of up to 30 days, demonstrating the improved delivery capabilities of the nanofibers (Hasanbegloo et al., 2023).

However, nanoparticles composed solely of single-component polymers have limitations, such as poor water solubility and susceptibility to clearance by the reticuloendothelial system. Amphiphilic block copolymers have garnered significant attention to enhance nanoparticle properties and achieve long-term therapeutic effects (Zhan et al., 2022). PEG as a hydrophilic moiety has been widely adopted, and nano-carriers based on the PEG-PCL architecture have become an essential strategy for increasing drug accumulation at specific target sites while minimizing non-specific drug uptake (Grossen et al., 2017). In line with this concept, Hongdan She and colleagues utilized ring-opening polymerization to synthesize methoxy polyethylene glycol-block-poly(ε-caprolactone) (mPEG-b-PCL) copolymers. Subsequently, they derived the polymer mPEG-b-PCL-DOX through orchestrated esterification and amidation reactions. *In vitro* experiments demonstrated that the IC50 of free DOX and mPEG-b-PCL-DOX NPs on HCT116 cells were 3.85 ± 0.16 μg/mL and 2.65 ± 0.29 μg/mL, respectively, highlighting the superior anti-tumor activity of the nanoparticles (Shen et al., 2021).

In contrast to monotherapy, combination therapy harnesses the synergistic effects of multiple drugs to achieve superior anti-tumor effects. Akanksha Behl and colleagues developed a multifunctional nano-carrier delivery system, PEG-PCL, capable of simultaneously delivering the chemotherapeutic drug Gemcitabine (GEM) and a MUC1 inhibitor. MUC1 is a transmembrane MUC found in human breast tumors due to its high overexpression. The MUC1 inhibitor disrupts the nucleus of human breast cancer cells, disrupts redox balance, and triggers DNA damage response. In vivo experiments, the average tumor volumes for Gem NPs, MUC1 inhibitor NPs, Gem-MUC1 inhibitor NPs, blank NPs, 5-FU, and sterile saline were approximately 828.75, 747.07, 473.75, 1055.14, 373.92, and 1119 mm^3^, respectively. Additionally, the NPs demonstrated strong tumor-targeting capabilities in the acidic tumor microenvironment, enhancing the efficacy of anti-cancer drugs both *in vitro* and *in vivo* (Behl et al., 2022). Furthermore, Jin et al. (2023) synthesized mPEG-b-PC, simultaneously loaded with PTX and sorafenib, achieving significant results with a tumor growth inhibition rate of up to 90.44%.

**FIGURE 2:**
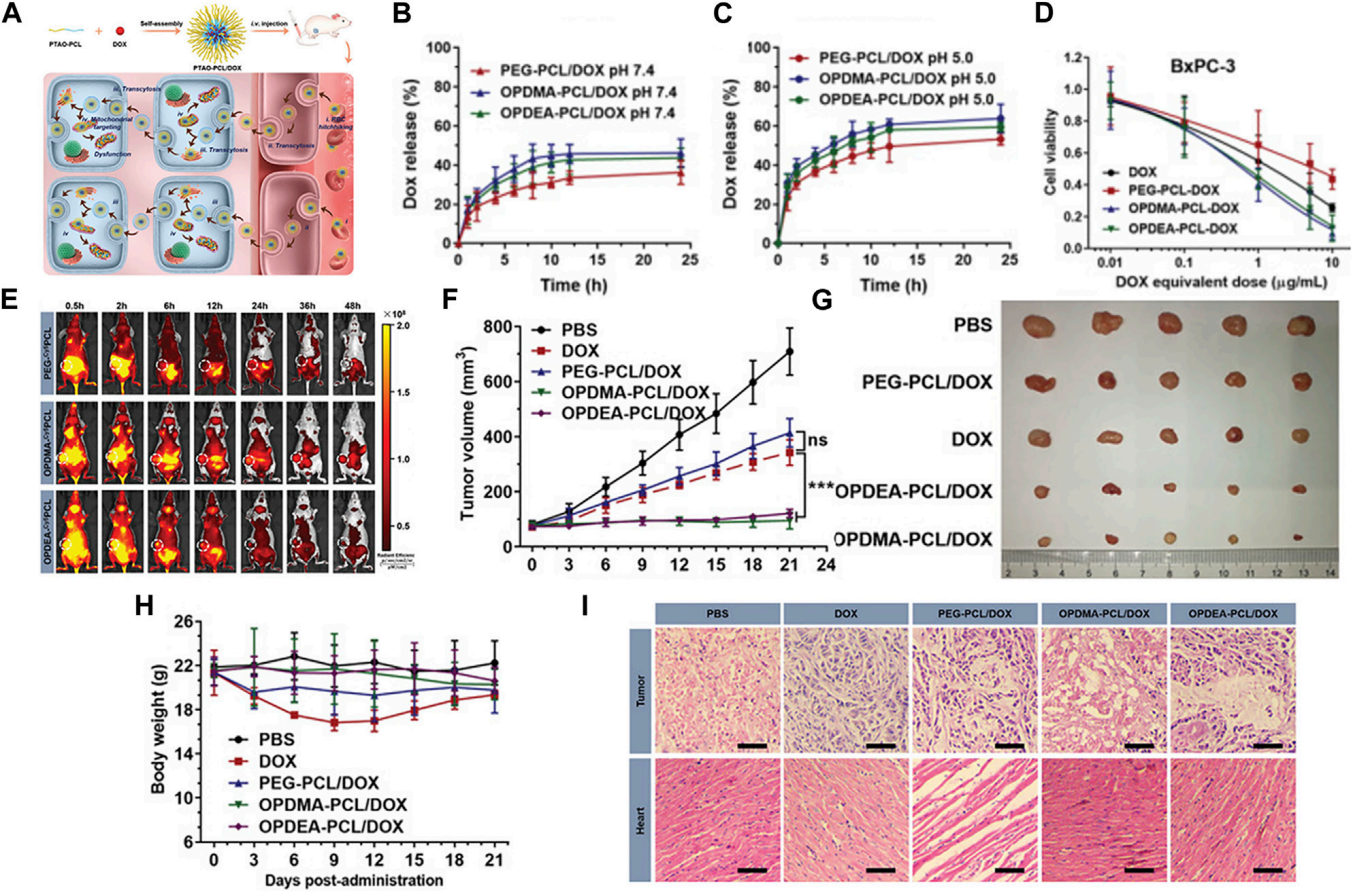
**(A)** Schematic illustration of PTAO-PCL micelles for cancer drug delivery. (a) The encapsulation of DOX into PTAO micelles self-assembled from PTAO-PCL block copolymers. (b) After intravenous injection, PTAO micelles i) circulate long in blood via red blood cell (RBC) hitchhiking and ii) attach on cell membranes to trigger transcytosis-mediated extravasation and iii) subsequent active tumor penetration. Inside tumor cells, a portion of PTAO micelles iv) target mitochondria and induce cell death. **(B)** The drug release profiles of the DOX‐loaded micelles at pH 7.4. **(C)** The drug release profiles of the DOX‐loaded micelles at pH 5.0. **(D)** The *in vitro* cytotoxicity of DOX‐loaded micelles against adherent BxPC‐3 and MCF‐7/ADR cells determined by the MTT assay (48 h treatment). **(E)** *In vivo* real‐time imaging of tumor‐bearing mice after a single intravenous injection of PEG‐^Cy5^PCL, OPDMA‐^Cy5^PCL, or OPDEA‐^Cy5^PCL (Cy5‐eq. dose of 0.5 mg kg^−1^). The tumor regions were circled in white. **(F)** Antitumor activities of DOX‐loaded PTAO‐PCL micelles against orthotopic MCF‐7/ADR tumors. Tumor growth curves of the mice. **(G)** Photographs of the tumors resected at the end of the experiment. **(H)** Body weight variation of the mice during the experiment. **(I)** Representative histological features of the tumors and hearts. The 10‐µm‐thick tissue sections were stained with hematoxylin‐eosin and observed using light microscopy. Scale bar: 50 µm. Reproduced with permission from ref Xiang et al. (2022). CC BY 4.0. Copyright 2022 The Authors.

In addition to using PEG, Jiajia Xiang, and colleagues discovered a versatile poly(tertiary amine oxide) (PTAO) as a superior alternative to PEG. PTAO-PCL/DOX exhibited enhanced tumor enrichment compared to PEG-PCL/DOX. Specifically, OPDMA-PCL/DOX and OPDEA-PCL/DOX micelles displayed DOX accumulation in tumors 2.7 and 2.3 times higher than PEG-PCL/DOX. This phenomenon could be attributed to PTAO-PCL micelles inducing extracellular transport, allowing PTAO-PCL/DOX to bypass the enhanced permeability and retention effect. Regarding tumor penetration, it was over 27 times more potent than PEG-PCL micelles. Furthermore, DOX-loaded PTAO micelles could target mitochondria, leading to mitochondrial dysfunction (Xiang et al., 2022) (See Figure 2 for further details).

Mitochondria produced ATP for ATP-binding cassette transporters like P-glycoprotein. They may possess mutations in mitochondrial DNA that contribute to multidrug resistance (MDR). So disrupting ATP synthesis and causing DNA damage within mitochondria could be an approach to surmount the multidrug resistance in tumor cells (Choi and Yu, 2014).

Furthermore, Zhang et al. (2022a) ingeniously enveloped PCL-PEG nanoparticles carrying respiratory syncytial virus (RSV) within red blood cell membranes to evade potential interactions with the immune system. This inventive fusion gave rise to a biomimetic nano-carrier named RSV-NPs@RBCm. Notably, this design exhibited remarkable potential for evading macrophage phagocytosis and demonstrated an extended circulation effect.

Most cancer cells highly express polyamine transport systems, considered promising tumor targeting sites. These sites can significantly enhance cellular uptake efficiency and boost cytotoxicity against cancer cells. Therefore, Zhang et al. (2022b) employed spermine (Spm) to modify PEG-PCL micelles, imparting them with strong targeting properties for carrying DOX. *In vivo*, experimental results demonstrated that micelles attached to the surface of PLGA microspheres greatly improved drug accumulation in the lungs and tumors. The combination of passive and active targeting mechanisms significantly enhanced the efficiency of DOX targeting.

**FIGURE 3:**
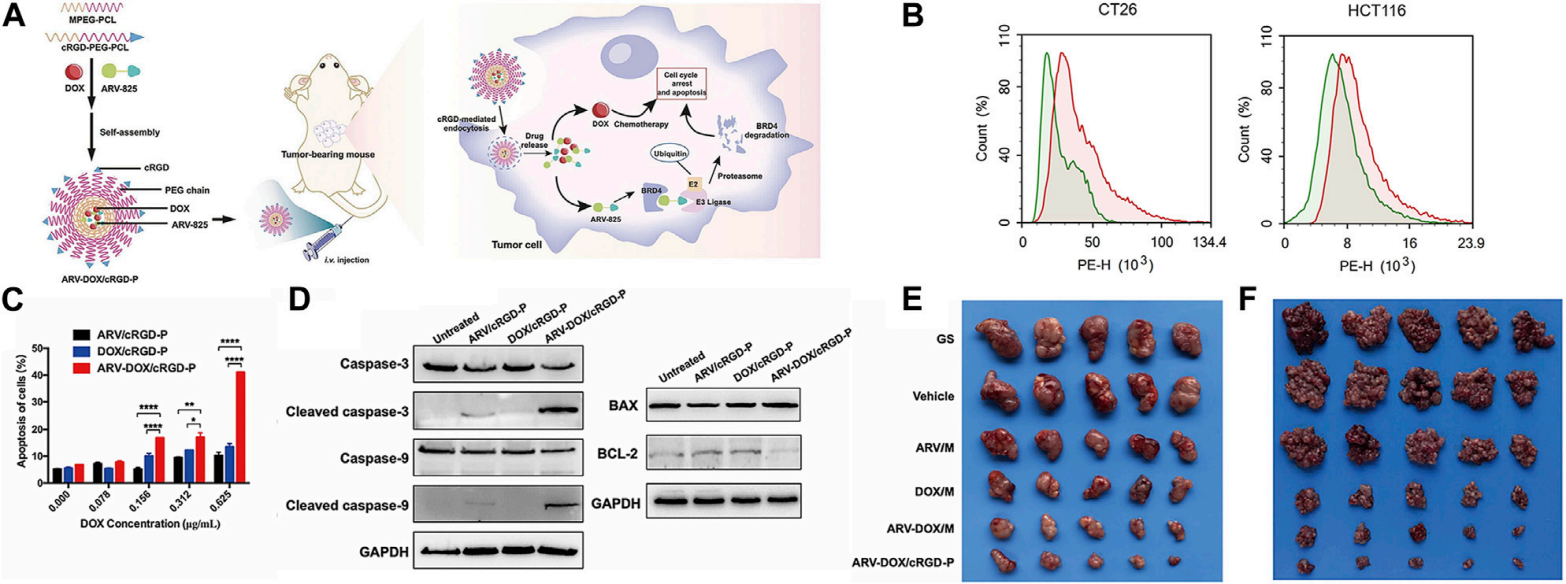
**(A)** Schematic illustration of the co-encapsulation of DOX and ARV-825 by cRGD-decorated nanoparticles, and the processes of drug delivery in colorectal tumor-bearing mice. Upon entering cells, ARV-825 binds to highly expressed BRD4, causing BRD4 degradation and promoting apoptosis together with DOX. **(B)** Cellular uptake comparison between ARV-DOX/M and ARV-DOX/cRGD-P nanoparticles. The green area indicated the ARV-DOX/M group and the red area indicated the ARV-DOX/cRGD-P group. **(C)** Percentages of apoptosis cells. (*n* = 3. * represents *p* < 0.05, ** represents *p* < 0.01, **** represents *p* < 0.0001). **(D)** CT26 cells were treated with ARV/cRGD-P (1.25 μg/mL), DOX/cRGD-P (0.625 μg/mL) or ARV-DOX/cRGD-P (equal amount) for 48 h, and apoptosis-related proteins were measured by Western blot. **(E)** ARV-DOX/cRGD-P complexes improved anti-tumor activity in a subcutaneous xenograft model. The subcutaneous CT26 tumor-bearing mice were intravenously administered with different formulations (GS, Vehicle, ARV/M, DOX/M, ARV-DOX/M, or ARV-DOX/cRGD-P). Tumor images after treatments. **(F)** ARV-DOX/cRGD-P complexes attenuated abdominal dissemination in colorectal cancer. Mice were intraperitoneally inoculated with CT26 cells and treated with GS, Vehicle, ARV/M, DOX/M, ARV-DOX/M, or ARV-DOX/cRGD-P. Images of tumor nodules. Reproduced with permission from ref He et al. (2023). CC BY 4.0. Copyright 2023 The Authors.

Previous studies have revealed significant expression of αvβ3 integrins in tumor tissues, and cyclo (Arg-Gly-Asp-D-Phe-Lys) (cRGD) has been shown to specifically bind to its receptors (Chou, 2010; Fang et al., 2017). Leveraging this knowledge, Yihong He and colleagues developed cRGD-PEG-PCL nanoparticles loaded with the chemotherapy drug DOX and the bromodomain-containing protein 4 (BRD4) degrader ARV-825. As depicted in Figure 3, cell uptake studies revealed that the red fluorescence intensity of the ARV-DOX/cRGD-P group was significantly higher than that of the ARV-DOX/M group, indicating superior targeting capabilities of the cRGD-P vector. Cell apoptosis experiments demonstrated that ARV-DOX/cRGD-P promoted cell apoptosis by activating the caspase signaling pathway and the BCL-11/BAX pathway in colorectal cancer cells, with a much more pronounced effect than other treatment groups. In subcutaneous tumor and peritoneal dissemination models, cRGD-PEG-PCL exhibited the most potent therapeutic effect. This underlined the excellent targeting and anti-cancer efficacy of this nanoparticle from various angles (He et al., 2023) (Refer to Figure 3 for further details).

### 2.2 Poly (lactic-co-glycolic acid) (PLGA)

PLGA stands out as one of the most successful advancements in drug delivery systems, which is known for its biocompatibility and flexibility to control particle polymer systems by changing chemical structure and molecular weight (Molavi et al., 2020). Its capacity to undergo hydrolysis within the body, ultimately breaking down into biodegradable monomers such as lactic acid and glycolic acid. This property not only ensures exceptional biocompatibility but also minimizes systemic toxicity. Consequently, PLGA is highly suitable for use as a carrier in drug delivery and as a crucial material in various biomedical applications (Kumari et al., 2010; Zhu et al., 2018). The details about the applications of PLGA in cancer chemotherapy are shown in Table 3.

**TABLE 3:** PLGA-based nanoparticle delivery systems investigated for treating cancers.

Author	Year	Nanoparticles system	Encapsulation efficiency (%)	Size (nm)	Drug loading (%)	IC50	Type of tumor	Chemotherapy drug	Reference
Hongqiao Cai et al	2021	MPGNPs	74.1	192	20	PANC-1 cells, 16.1 nM	Pancreatic cancer	Gemcitabine	Cai et al. (2021)
Xiaozheng Zhao et al	2021	NS-TAX@Lipo-VAC	—	200	5	—	Pancreatic cancer	TAX	Zhao et al. (2021)
Ru Zhang et al	2022	DOX/FA-HASS-PLGA	83.25 ± 0.45	307.47 ± 1.50	21.1 ± 0.1	—	Breast cancer	DOX	Zhang et al. (2022c)
Meng Wang et al	2022	PG@KMCM	—	117.8 ± 54.5	82.8	—	Pancreatic cancer	Gemcitabine	Wang et al. (2022)
Razan B. Al-Humaidi et al	2022	paclitaxel-PLGA-NPs	59	85.54 ± 0.6427	41.41	MCF-7 cells, 39.41 ± 1.33 nM	Breast cancer	PTX	Al-Humaidi et al. (2022)
Reem M. Gahtani et al	2023	5-FU-PLGA	≥90	200	1	—	Lung cancer	5-FU	Gahtani et al. (2023)
Fakhrossadat Emami et al	2023	DRT-DTX-PLGA	71.9 ± 1.2	124.2 ± 1.1	2.5 ± 0.8	—	Glioblastoma and lung cancer	DTX	Emami et al. (2023)
Yue Li et al	2023	CCMNPs	74.42	94.13	4.16	NCI-H460 cells, 3.02 μg/mL	Lung cancer	PTX	Li et al. (2023)
Huai-An Chen et al	2023	HA/PMNPc	—	300	18	U87 cells, 0.297 μg/mL	Glioblastoma	CDDP	Chen et al. (2023)
Dasharath Chaudhari1 et al	2023	PTX- ADN-PEG-PLGA	79.26 ± 2.52	135 ± 12	7.5	4T1 cells, 3.16 μg/mL	Breast cancer	PTX	Chaudhari et al. (2023)

Due to its excellent biocompatibility, many researchers have considered utilizing PLGA as a viable option for drug delivery (Shive et al., 1997). Reem M. Gahtani et al. prepared PLGA nanoparticles loaded with 5-FU. *In vitro* experiments revealed a biphasic release pattern of 5-FU from these nanoparticles, with an initial rapid release followed by a slow and steady release. When cells were treated with a 5-FU solution, cell viability decreased by 70%, whereas 5-FU-PLGA-NPs containing the same drug dose induced nearly 100% cell toxicity. This suggested that the delivery system may significantly enhance intracellular drug accumulation and improve therapeutic efficacy (Gahtani et al., 2023).

**FIGURE 4:**
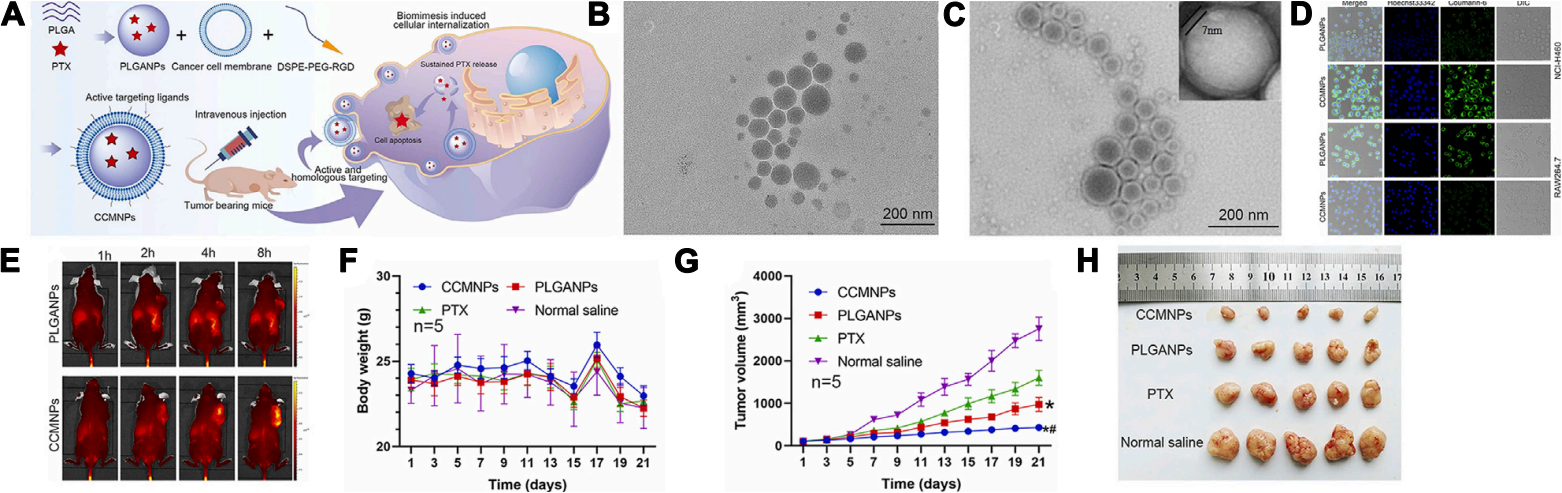
**(A)** Assembly of CCMNPs, injection in mice, and *in vivo* drug release. **(B)** TEM image of PLGANPs. **(C)** TEM image of CCMNPs. **(D)** Laser confocal microscopic images of NCI-H460 human lung cancer cells and mononuclear macrophages of RAW264.7 mice. **(E)** *In vivo* distribution over time after tail vein injection of PLGANPs and CCMNPs in BALB/C nude mice. **(F)** Broken line diagram of weight change in nude mice bearing tumor. **(G)** Broken line diagram of tumor volume changes in nude mice bearing tumor. **(H)** Experimental results of tumor growth inhibition after administration, *n* = 5. Reproduced with permission from ref Li et al. (2023). CC BY 4.0. Copyright 2023 The Authors.

Nanoparticles possess a drawback in that the reticuloendothelial system recognizes them as foreign particles and are consequently partially cleared by immune cells. However, this limitation can be effectively addressed by employing biomimetic nanoparticles cloaked with natural cell membranes, which remarkably enhance the targeted delivery of drugs to specific cells (Li R. et al., 2019). In light of this, Hongqiao Cai et al. devised an innovative strategy involving macrophage membrane-coated nanoparticles (MPGNPs) that were loaded with gemcitabine and encapsulated within PLGA nanoparticles. This approach aimed to mitigate drug toxicity while simultaneously enhancing drug accumulation within tumors (Cai et al., 2021). Furthermore, building upon the concept of cell membrane coating, Yue Li et al. introduced the incorporation of tumor cell membrane (CCM) onto PLGA nanoparticles to achieve immune evasion. Comparative investigations between PLGANPs and PTX injection disclosed that CCMNPs showcased 1.3- and 2.0-fold tumor suppression rates in xenograft nude mice models, respectively (Li et al., 2023) (Refer to Figure 4 for further details).

Sometimes, the EPR effect is highly variable, and the frequent occurrence of low EPR, especially in clinical tumors, compromises the delivery of EPR-dependent nanoparticles (Tietjen and Saltzman, 2015). The researchers used tumor cell-specific ligands as targets to confer active targeting to the delivery system, aiming to improve the limitations of the EPR effect. In recent years, adenosine (ADN) receptors have emerged as pivotal mediators in tumor growth and progression. Studies conducted by Swami et al. (2015) have revealed that ADN could effectively function as a targeting ligand, directing delivery systems toward specific cancer cells. Chaudhari et al. (2023) harnessed ADN as a targeting ligand while utilizing PEG as a linker to augment hydrophilicity. They made PLGA-PEG-ADN nanoparticles loaded with paclitaxel for anti-cancer therapy. The results indicated that The ADN modification over PLGA NPs rendered higher particle internalization, resulting in a 3.5-fold reduction in IC50 values in TNBC cells. Further, the ADN modification allowed particles to exhibit a higher apoptosis index in TNBC cells when compared to non-modified PLGA NPs and the free PTX group. This demonstrated the superior anti-cancer performance of the nanoparticles containing targeting ligands.

In addition to targeting ligands, it is possible to achieve chemo-photothermal combined therapy for cancer by co-loading chemotherapy drugs and photothermal agents. Chen et al. (2018) prepared hyaluronic acid (HA)-modified PLGA nanoparticles, where HA exhibited targeting by binding to CD44 receptors on the surface of tumor cells. Alongside encapsulating the chemotherapy drug CDDP, they simultaneously loaded oleic acid-coated iron oxide magnetic nanoparticles (IOMNP) with photothermal properties, resulting in HA/PMNPc nanoparticles. The IOMNP served as a photothermal agent for photothermal cancer therapy when exposed to near-infrared light. On the one hand, targeted drug delivery increased the drug’s therapeutic effect On the other hand, the nanoparticles exhibited a hyperthermic effect upon short-term near-infrared light irradiation, further enhancing cell apoptosis through photothermal effects. Results demonstrated that HA/PMNPc nanoparticles increased intracellular uptake through active targeting and improved drug release rates in the acidic intracellular environment. Additionally, their cytotoxicity was enhanced, with an IC50 value only at 46% of the free drugs. In *in vivo* experiments, mice injected with HA/PMNPc nanoparticles exhibited the slowest tumor growth rate and longest survival time. In conclusion, the dual-targeting ability and chemophotothermal treatment capabilities provided by HA/PMNPc hold significant potential for cancer therapy (Chen et al., 2023).

Dual-receptor targeting nanoparticles containing two different targeting agents have garnered widespread attention due to their potential for higher cellular selectivity, cellular uptake, and cytotoxicity against cancer cells. Fakhrossadat Emami and colleagues functionalized PLGA nanoparticles with anti-EGFR antibodies and anti-PDL1 antibodies, encapsulating DTX to create DRT-DTX-PLGA nanoparticles. The results showed that compared to other formulations, treatment with DRT-DTX-PLGA significantly reduced the cell viability of human glioblastoma cells U87-MG and human non-small cell lung cancer cells A549, with survival rates of 25.1% ± 5.3% and 20.6% ± 7.8%, respectively. In both cell lines, the cytotoxic effects of DRT-DTX-PLGA were significantly higher than those of NT-DTX-PLGA and free DTX, indicating a substantial synergistic enhancement of intracellular uptake by the dual ligand nanoparticle system (Emami et al., 2023).

Wang et al. (2022) had also developed dual-targeting nanoparticles known as PG@KMCM. The results showed that these nanoparticles could effectively reprogram the tumor microenvironment, killing pancreatic cancer cells and enhancing the overall therapeutic potential.

**FIGURE 5:**
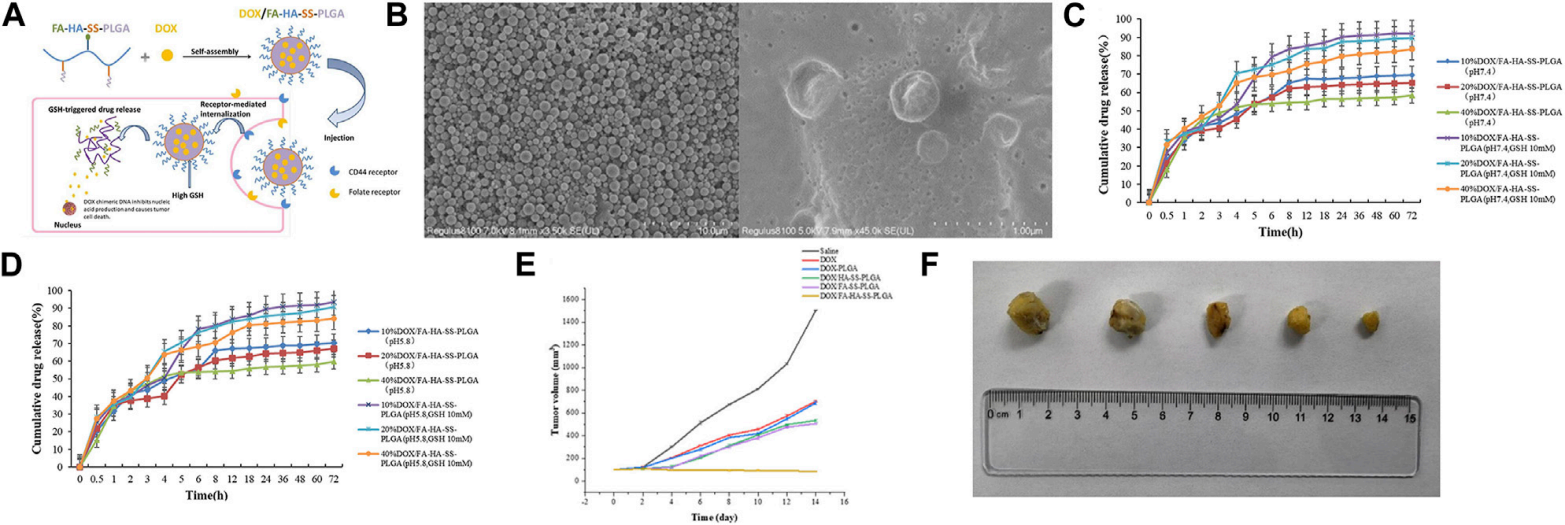
**(A)** Illustration of self-assembly, tumor aggregation, and intracellular release of doxorubicin hydrochloride (DOX)/FA-HA-SS-PLGA micelles. **(B)** Scanning electron microscopy (SEM) micrographs of FA-HA-SS-PLGA and DOX/FA-HA-SS-PLGA. **(C)** The *in vitro* release curve of DOX in PBS buffer at pH 7.4. **(D)** The *in vitro* release curve of DOX in PBS buffer at pH 5.8. **(E)** The tumor growth volume after treatment with saline, DOX, DOX-PLGA, DOX/HA-SS-PLGA, DOX/FA-SS-PLGA, and DOX/FA-HA-SS-PLGA. **(F)** The tumor morphology after treatment with saline, DOX, DOX-PLGA, DOX/HA-SS-PLGA, DOX/FA-SS-PLGA, and DOX/FA-HA-SS-PLGA. Reproduced with permission from ref Zhang et al. (2022c). CC BY 4.0. Copyright 2022 The Authors.

However, there was a problem in previous studies: the drug encapsulated in the carrier cannot be released after tumor cells ingest this dual-target drug delivery system. Therefore, Zhang et al. (2022c) designed dual-targeting nanoparticles, DOX/FA-HASS-PLGA, where folic acid (FA) can bind to overexpressed folate receptors on cancer cell surfaces, and hyaluronic acid (HA) can bind to overexpressed CD44 receptors on cancer cell surfaces. Moreover, they used disulfide bonds to connect the HA hydrophilic shell to the PLGA hydrophobic core. Because glutathione (GSH) levels in tumor cells were 7–10 times higher than in normal tissues, this highly reducing environment led to rapid breakage of micelles that reached the tumor site via thiol-disulfide bond exchange, which allowed for rapid drug release. In experiments with tumor-bearing mice, the DOX/FA-HA-SS-PLGA group exhibited the highest survival rate, smallest tumor volume, and significantly extended average survival time compared to other control groups. These results further illustrated that DOX/FA-HA-SS-PLGA possessed the most effective anti-cancer properties, and these dual-targeting reducible drug-loaded micelles had a promising therapeutic effect on tumors (Refer to Figure 5 for further details).

### 2.3 Polylactic acid (PLA)

PLA is a lactic acid derivative derived from renewable sources such as wheat, straw, corn, and sorghum (Rydz et al., 2014). Recent research has shown that it can also be extracted from agricultural waste materials like sugarcane bagasse and olive pits (Cox et al., 2023; Haokok et al., 2023). Its remarkable biodegradability distinguishes PLA, as it undergoes degradation within the body into lactic acid monomers that participate in the human tricarboxylic acid cycle, ultimately breaking down into CO_2_ and water (Kumari et al., 2010). It has been widely used in drug delivery due to its biodegradability and tunable mechanical properties (Lee et al., 2016). The details about the applications of PLA in cancer chemotherapy are shown in Table 4.

**TABLE 4:** PLA-based nanoparticle delivery systems investigated for treating cancers.

Author	Year	Nanoparticles system	Encapsulation efficiency (%)	Size (nm)	Drug loading (%)	IC50	Type of tumor	Chemotherapy drug	Reference
Jianhua Chen et al	2021	DTX-mPEG-PLA	—	100	—	—	Sarcoma	DTX	Chen et al. (2021a)
Sungho Lee et al	2021	PTXx@Hap	—	80	—	—	Breast cancer	PTX	Lee et al. (2021)
Jamie K. Hu et al	2021	PLA-HPG	—	200–300	—	—	Skin cancer	Camptothecin	Hu et al. (2021)
Mohd Anees et al	2022	mPEG-PLA and LA-pluronic L-61-PLA	83.3 ± 4.6/94.7 ± 2.2 DOX/PIRA	130.5 ± 2.4	3.19 ± 0.13/3.61 ± 0.06 DOX/PIRA	MDA-MB 231 cells 1.378 ± 0.336/0.293 ± 0.075 nM DOX/PIRA	Breast cancer	DOX/PIRA	Anees et al. (2022)
Neha Mehrotra et al	2023	NAV/DCB NPs	40–70	90–145	—	—	Breast cancer	Decitabine	Mehrotra et al. (2023)

Qin et al. (2019) once employed ultrasound emulsification to merge PLA, with chitosan, resulting in nanoparticles loaded with 5-fluorouracil and irinotecan. *In vivo* experiments also unveiled its capacity to suppress the growth of cancers, outperforming intravenous injection.

However, nanoparticles can result in challenges such as poor solubility. To address issues related to dissolution and stability of nanoparticles, researchers have implemented strategies involving the chemical crosslinking of hydrophilic polyethylene glycol (Im et al., 2021).

Therefore, Chen et al. (2021a) employed the emulsion solvent diffusion method to synthesize DTX-mPEG-PLA nanoparticles designed for sarcoma treatment. Incorporating a PEG shell could enable prolonged circulation and facilitate tumor targeting through the EPR effect. The study results showed that due to the nanoparticles’ good pharmacokinetic properties, DTX NPS showed a tumor inhibition rate of 94.66% in a hormonal mouse model, which was 1.24 times higher than that of DTX injection. These findings emphasize the promise of mPEG-PLA nanoparticles in advancing drug delivery.

**FIGURE 6:**
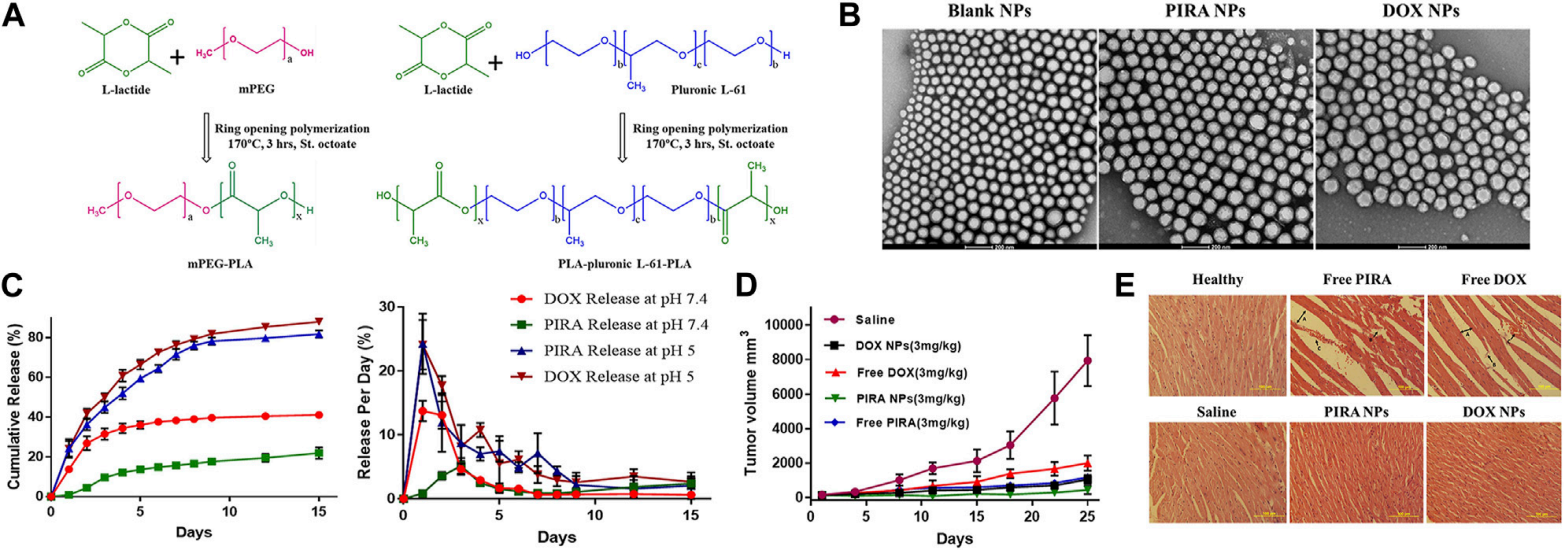
**(A)** Schematic representation of ring opening polymerization of L-lactide using polymerizing initiators mPEG and pluronic L-61. **(B)** HR-TEM images of PLA-based hybrid block copolymeric NPs. The scale bar represents 200 nm. **(C)** *In-vitro* release profile of PIRA/DOX from PLA-based hybrid block copolymeric NPs at pH 7.4 and pH 5. **(D)** Change in tumor volume of mice treated with free drugs, drug-loaded NPs, and saline throughout the study period. **(E)** Histopathological images of cardiac tissue of all treated/untreated mice. The scale bar represents 100 µm. Reproduced with permission from ref Anees et al. (2022). CC BY 4.0. Copyright 2022 The Authors.

To enhance the hydrophobicity of the diblock copolymer core and make the nanoparticles denser, Mohd Anees et al. blended a pentablock copolymer PLA-pluronic L-61-PLA with mPEG-PLA as a hybrid system to prepare PIRA encapsulated NPs. *In vivo* experiments, the tumor regression rates for free DOX and free PIRA treatment in mice were 74.74% ± 4.5% and 85.07% ± 1.6%, respectively. However, the tumor regression rates increased to 86.65% ± 2.6% and 94.36% ± 2.3% when using DOX NPs and PIRA NPs, respectively. Significantly, the use of polymers did not induce unnecessary non-targeted cardiac toxicity or myocardial atrophy (Anees et al., 2022) (Refer to Figure 6 for further details).

**FIGURE 7:**
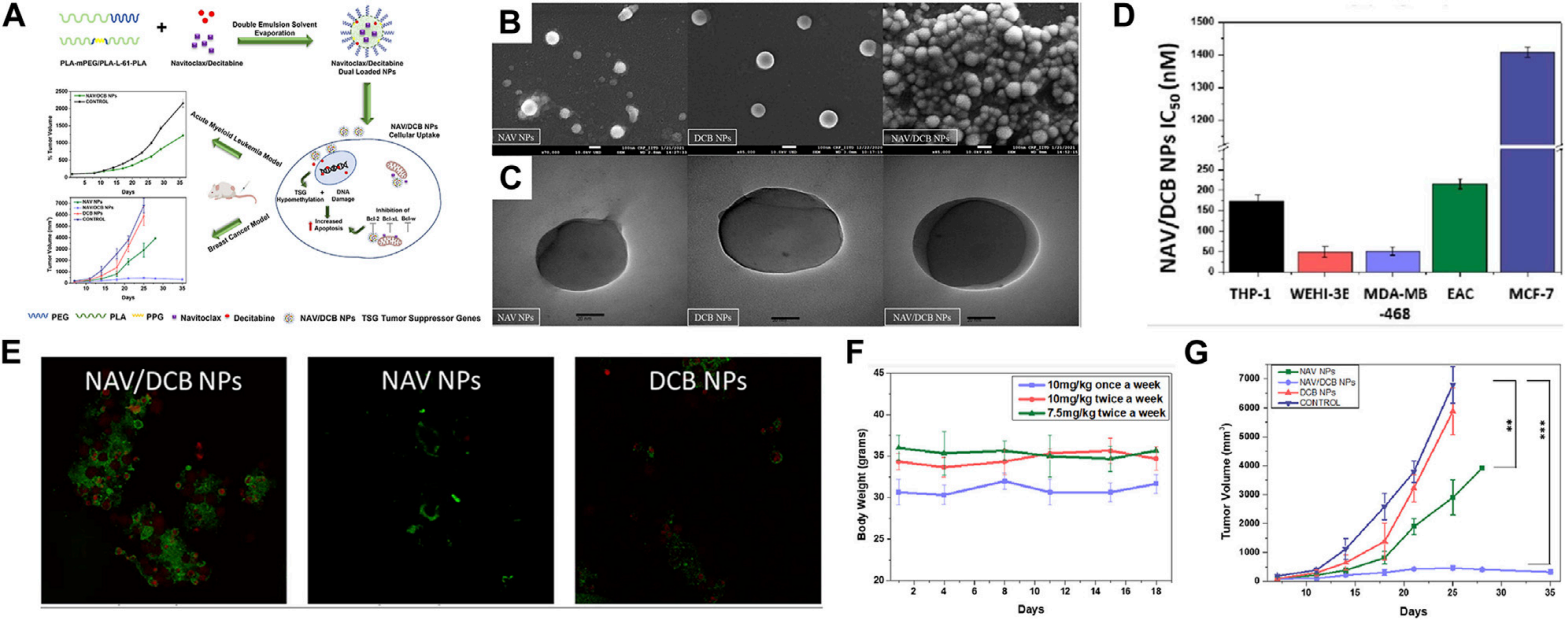
**(A)** Schematic representation of concomitant delivery of BH3 mimetic navitoclax and DNA methyltransferase inhibitor decitabine using polylactic acid hybrid block copolymeric nanoparticles. Upon entry of nanoparticles into cells, navitocla inhibits BCL expression and decitabine causes DNA damage, which together lead to apoptosis. The results showed potent synergistic cytotoxicity against both acute myeloid leukemia and breast cancer cell lines *in vitro*. **(B)** FE-SEM images of NAV/DCB single and dual NPs. **(C)** HR-TEM images of NAV/DCB single and dual NPs. **(D)** IC50 values for NAV/DCB NPs on various AML and breast cancer cell lines. **(E)** Confocal microscopy in EAC cells post 48-h exposure to 100 nM NAV/DCB single and dual NPs. **(F)** *In vivo* dose tolerability study for NAV/DCB dual NPs in healthy BABL/c for three dosing schedules. **(G)** Tumor inhibition study using NAV/DCB single and dual NPs for syngeneic breast cancer model. Reproduced with permission from ref Mehrotra et al. (2023). CC BY 4.0. Copyright 2023 The Authors.

Mehrotra et al. (2023) also employed a hybrid-block copolymer nanoparticle system, PLA-mPEG/PLA-L61-PLA NPs, to simultaneously deliver the chemotherapy drug navitoclax and decitabine (DCB) for combined cancer therapy. Decitabine is a chemotherapy drug, while navitoclax is one of the first-generation pan-Bcl-2 inhibitors that have demonstrated potent activity in clinical trials against certain solid tumors. In cell experiments, the NAV/DCB dual-drug-loaded NPs significantly reduced the IC50 values compared to NAV/DCB dual-drug-loaded NPs alone, indicating a synergistic mechanism of action. In animal experiments, the NAV/DCB dual-drug-loaded NPs exhibited a significant tumor growth inhibition effect in a xenograft tumor model. Compared to the control group, the treatment group saw a 43.6% reduction in tumor size, once again demonstrating the synergistic effect of dual-drug loading (Refer to Figure 7 for further details).

In addition to using PEG to form hydrophilic shells, research has reported using other polymer molecules as hydrophilic shells. Hu et al. (2021) developed a bioadhesive nanoparticle (BNP) drug delivery system composed of biodegradable polymer, poly(lactic acid)-hyperbranched polyglycerol (PLA-HPG), encapsulating camptothecin (CPT). The surface chemistry of HPG molecules was altered by treatment with sodium periodate, converting adjacent diols into aldehydes. Aldehydes can form strong covalent bonds with amines on the surface of tumor cells and extracellular matrix proteins. In *in vivo* experiments, the results showed that after 10 days of injection of BNP-CPT, approximately 50% of CPT was still retained in the tumor, whereas CPT was undetectable in tumors injected with free CPT. BNP-CPT also significantly reduced tumor burden, with some established tumors (about 20%) showing histological cure following BNPCPT treatment.

Apart from targeting specific receptors, researchers have also capitalized on the distinct pH levels surrounding tumors to enhance drug delivery outcomes. Lee et al. (2021) adopted an approach to fabricate poly lactic acid/hydroxyapatite (PLA/HAp) core-shell nanoparticles loaded with PTX. Hydroxyapatite maintains stability under neutral pH conditions yet dissolves within acidic environments. This pH responsiveness enabled its dissolution in the acidic milieu characteristic of cancer cells, thus facilitating drug release. The outcomes revealed persistent cytotoxic effects on 4T1 cells for a duration of up to 48 h, signifying its potential as a drug carrier for tumor inhibition.

### 2.4 Other polyester

The design of thermoplastic polyesters (e.g., PLA) often requires the use of toxic initiators, catalysts, or solvents. So, catalyst-free thermal polyesterification has recently emerged as a potential strategy (Tham et al., 2016). Among them, Poly(Glycerol Sebacate) (PGS) can be formed by a polycondensation reaction of two monomers, glycerol, and sebacic acid, both of which are biocompatible and have been approved by the FDA.

In 2022, Massironi et al. (2022) prepared curcumin-loaded PGS-NPs by nanoprecipitation. The results showed that the PGS-NPs had good biostability over 14 days. The IC 50 value of curcumin-loaded PGS-NPs at 72 h (15.95 µM) was significantly lower than that of free curcumin (21.27 µM) suggesting a higher cytotoxic effect of curcumin-loaded PGS-NP. It suggested that curcumin-loaded PGS-NPs may represent a possible adjuvant therapy for treating cancer cells.

## 3 Discussion

### 3.1 The mechanisms of nanoparticles in cancer treatment

It was first thought that nanoparticles could passively extravasate into solid tumors through the porous vascular system and reside within the tumor to achieve accumulation, a phenomenon known as the enhanced permeability and retention (EPR) effect, a consensus that persisted for many years (Maeda, 2012).

But in recent years different discoveries have been made, Liu et al. (2019) found that nanoparticles could also enter tumors through an active transcellular transport process, and that transcytosis may be an important mechanism for cancer nanodrugs. It included receptor-mediated transcytosis (RMT), absorptive-mediated transcytosis (AMT), and bulk-phase or fluid-phase transcytosis (FPT) (Li and Kataoka, 2021).

Al-Humaidi et al. (2022) produced paclitaxel-PLGA-NPs with a particle size of 85.5 nm using the modified nanoprecipitation method. Through the use of different endocytosis inhibitors, they demonstrated that macropinocytosis was the primary endocytosis pathway for the paclitaxel-PLGA-NPs.

Sindhwani et al. (2020) also showed that nanoparticle entry into tumors is an active process rather than passive transport. These findings provide important fundamental theories and research directions for further advancement of nanoparticle-based drug delivery systems.

### 3.2 Effect of nanoparticle shape on properties

The shapes of nanoparticles can be broadly categorized as spherical and non-spherical, such as filamentous, discoidal, hemispherical, and worm-like, among others. It has been recognized as a key factor influencing cellular uptake, circulation time, biodistribution, and cancer drug delivery (Truong et al., 2015).

Compared to spherical nanoparticles, non-spherical shape hinders the uptake of microparticles by macrophages, with a negative correlation between uptake rate and aspect ratio, which prolongs the residence time of the nanoparticles in the bloodstream and increases their chances of reaching the target site (Florez et al., 2012; Mathaes et al., 2014). Non-spherical nanoparticles are also better than spherical nanoparticles in terms of tumor extravasation, on the one hand, it has a long circulation time in the blood, and on the other hand, it has a higher surface adhesion interaction area than spherical particles (Cooley et al., 2018).

Although non-spherical particles can improve cytotoxicity, alter biodistribution, and improve *in vivo* anti-tumor efficacy. However, the design of non-spherical particles for degradable polymers still faces many difficulties (Jindal, 2017).

In addition to nanoparticles of specific sizes and shapes, size- and shape-transformable nanoparticles have emerged as a promising strategy for tumor theranostics. But it also means their designs are more complex (Chen et al., 2021b).

### 3.3 The shell materials

As nanoparticles circulate within the body, they encounter challenges in immune system clearance mechanisms (Fu et al., 2021). In order to minimize RES clearance and prolong blood circulation time, various shell materials have been used to shield their surfaces and achieve a stealth effect, which prevents nonspecific protein adsorption and subsequent phagocytosis. Wen et al. (2023) provided a detailed review of invisible nanocarriers and proposed the concept of “pseudo-stealth effect.”

Among the various stealth shell materials, the most frequently employed is PEG (Cho et al., 2016; Liang et al., 2017; Menconi et al., 2021). Research on polyethylene glycol to extend the circulation time of liposomes in the bloodstream dates back to the 1990s (Klibanov et al., 1990). However, polyethylene glycolization prevents interaction with diseased cells, a problem known as the “PEG dilemma” (Hatakeyama et al., 2011). In addition to PEG, researchers have also identified other phospholipid-binding zwitterion that can enhance tumor permeability and prolong blood circulation, such as poly(2-(N-oxide-N, N-diethylamino)ethyl methacrylate) (OPDEA) (Chen S. et al., 2021).

People have also utilized biomimetic methods, such as using cell membrane coatings, which can make nanoparticles look more like their own cells, thus evading removal by the immune system (Chen S. et al., 2021; Guo et al., 2022). Brenner et al. (2018) have also utilized red blood cell (RBC)--hitchhiking (RH) to increase uptake of nanoparticles in organs. Zhang et al. (2020) also produced albumin-based nanoparticles to increase circulation time and reduce the toxic side effects of free drugs.

### 3.4 Stimuli-responsive polyester

Nanodrugs are usually released prematurely before the nanocarriers reach the target lesions. Therefore, the application of stimuli-responsive nanomaterials for drug delivery has received increasing attention. Stimuli-responsive nanomaterials can be categorized into three classes, endogenous stimuli-responsive materials, exogenous stimuli-responsive materials, and multi-stimuli-responsive materials (Li L. et al., 2019).

Endogenous stimuli-responsive materials mainly include pH, enzyme, and redox-responsive materials. Tumor tissues exhibit a slightly acidic extracellular pH of around 6.5, while normal tissues typically range from pH 7.2 to 7.4 (Singhvi et al., 2019). Consequently, researchers have harnessed these environmental variations to develop pH-sensitive drug delivery systems (Zheng et al., 2020; Qian et al., 2021). Nanocarriers can also utilize the different pH gradients within cellular components to achieve precise drug release (Zhang et al., 2014).

Enzyme-responsive delivery systems have also received increasing attention. Li et al. (2021) exploited the MMP overactivation in tumor-associated tissues to design enzymatically transformable polymersomes-based nanotherapeutics to guide the co-delivery of colchicine and marimastat. It not only exposes the guanidine moiety for improving tissue/cell targeting to enhance bioavailability but also to differentially release drugs.

Glutathione levels are usually elevated in tumor cells, which leads to a higher reducing environment. Therefore, nanoparticles based on redox-responsive drugs targeting cancer cells have also been extensively studied (Park et al., 2015).

Exogenous stimulus-responsive materials such as light (Chen Y. et al., 2021), ultrasound (Papa et al., 2017; Huang et al., 2023), and magnetic field (Garcia-Garcia et al., 2020) responsive materials can also be applied to achieve accurate drug release at the tumor site. The combined application of multiple stimulus-responsive materials has also received attention. In conclusion, the stimuli-crosslinking strategy shows promising potential for cancer treatments (Xue et al., 2022).

## 4 Current limitations

Ideally, nano drug delivery systems should be low or even non-toxic, have good drug encapsulation efficiency, be capable of controlled release, and continuous delivery, and be easy to perform clinically. Unfortunately, current technologies cannot simultaneously fulfill all of these requirements (Liu et al., 2023).

Despite significant advances in biodegradable polyesters, many shortcomings remain. PLA suffers from shortcomings (low-ductility and toughness, glass transition and heat distortion temperature, rate of crystallization; high sensitivity to moisture and fast degradation by hydrolysis, etc.) (Murariu and Dubois, 2016). PCL has low mechanical strength, an insufficient number of cellular recognition sites, poor bioactivity, and hydrophobicity (Homaeigohar and Boccaccini, 2022). PLGA also faces drawbacks such as low drug loading, high production cost, and difficulty in large-scale production (Lu et al., 2023).

Furthermore, most nanoparticles were still in the cellular and animal experimental stages. Some have entered clinical trials, but the results were often unsatisfactory. People realized that the EPR affected works in rodents, but its role in humans needed to be further verified (Nichols and Bae, 2014; Danhier, 2016). They still have a long way to go before they can be used in clinical applications (Wilhelm et al., 2016; Ouyang et al., 2020).

First, the safety issue is of utmost concern. The entry of nanoparticles into the blood circulation may cause adverse effects, such as inflammation, cell cycle alteration, oxidative stress, DNA damage, etc. (Ciappellano et al., 2016). Whether adequate clearance through the glomerular filtration membrane is possible deserves further investigation. In addition, non-specific accumulation in normal tissues may also occur, resulting in further adverse effects (Rehman et al., 2022).

In addition, acidic products were observed during the degradation of polyester, which can lead to an inflammatory response. Polycarbonate is superior to polyester in this respect, as it does not produce acidic products during degradation (Yu et al., 2021).

Nanoparticles also face low drug loading rates, with most of the nanoparticles currently approved by the FDA having no more than a 20% drug loading rate. This means that excessive carrier material may be required to achieve a therapeutic effect, which needs to be further verified to see if this will further exacerbate its potential toxicity. In addition, to increase stability, coatings such as PEG are added to prevent premature uptake by macrophages, but this can also lead to a decrease in encapsulation rate when new materials are added. Consequently, this leads to difficulties in applying it to clinical (Liu et al., 2020; Et et al., 2021).

In addition, biodegradable polyester nanoparticles face problems such as complicated fabrication and high cost (James et al., 2016). These hinder its mass production and become a hindrance to clinical applications.

## 5 Conclusion and outlook

As drug delivery carriers, biodegradable polyesters have many good properties, such as increasing the solubility of hydrophobic drugs, improving drug efficacy, prolonging drug action time, and improving drug bioavailability (Janrao et al., 2023). This review summarizes the recent applications of biodegradable polyester-based nano-drug delivery systems over the past 3 years.

In recent years, polyester-based delivery systems have been increasingly studied, ranging from individual nanoparticles to amphiphilic block copolymers. Subsequently, their surfaces are modified to avoid phagocytosis by the immune system. In addition, the drugs carried have evolved from single chemotherapeutic agents to combinations of multiple drugs. Some researchers have also carried nucleic acids (Zhao et al., 2018; Zhao et al., 2023), SiRNA (Xu et al., 2018), and immunomodulatory agents (Narmani et al., 2023), yielding desirable results.

A polyester-based polymer micelle known as Genexol-PM has received marketing approval in South Korea. A Phase III clinical trial has revealed that it exhibited non-inferior and even superior clinical efficacy when compared to standard paclitaxel in patients with a manageable safety profile in patients with metastatic breast cancer. This represented a milestone for polymeric nanomedicines toward clinical translation (Kim et al., 2001; Park et al., 2017; Yi et al., 2018).

However, for the other nanoparticles, there is still a need for further safety and toxicology testing of nanoparticles to ensure their benefits outweigh their harm to the human body. It is also essential to find ways to improve its drug loading rate for more efficient treatment, which will also reduce the impact of nanoparticles themselves on the human body.

The process of making nanoparticles also needs to be further simplified to ensure its robustness. This will further increase the possibility of it being promoted in the clinic treatments.
